# Esketamine for Treatment-Resistant Depression: A Case Series and Literature Review

**DOI:** 10.7759/cureus.92165

**Published:** 2025-09-12

**Authors:** Muhammad Abbas, David B Adams, Jack Noto, Renzmark D Vallesteros, Talitha West

**Affiliations:** 1 Psychiatry, Hackensack Meridian School of Medicine, Nutley, USA; 2 Psychiatry, Hackensack Meridian Ocean Medical Center, Brick, USA

**Keywords:** case report series, depression, esketamine, literature review, treatment-resistant depression

## Abstract

Management of treatment-resistant depression (TRD) is complex and frustrating for patients, requiring several treatment courses before (or without) achieving remission. This investigation aims to highlight and analyze real-world instances of using esketamine for patients with TRD who have failed electroconvulsive therapy (ECT).

Four case reports from a single Hackensack-Meridian care center are described in detail. They, along with a literature review, were then used to drive a narrative discussion on the potential of esketamine as a TRD therapy. Of the four cases analyzed, three patients experienced significant improvement in symptoms. However, largely due to side effects, only one case resulted in effective long-term treatment using esketamine.

Given that all four patients previously failed ECT, these results are encouraging. Even a humble success rate may inspire hope in a patient population that is largely considered intractable using current methodology. This study found that esketamine is a viable alternative for some patients suffering from TRD. This even remains true in some of our patients who have failed ECT - one of the more effective, more aggressive therapies for TRD.

## Introduction

In 2021, the National Institute of Mental Health reported that 14.5 million adults in the U.S. experienced at least one episode of major depression within that past year [1]. Treatment-resistant depression (TRD) is a label given to patients with depressive disorders who do not respond to at least two antidepressants administered at adequate doses and duration. It is a label that does not currently benefit from universal diagnostic criteria, rather, it is a widely used term in the clinical literature. While estimates of TRD prevalence vary, one recent study estimated that the 12-month prevalence of TRD in the US is over 2.8 million individuals [2].

Several treatment options are available for TRD. The American Psychiatric Association [3] recommends augmenting initial pharmacotherapy as-needed with medications such as lithium, thyroid hormone, anticonvulsants, or second-generation antipsychotics. Furthermore, a 2018 Cochrane meta-analysis [4] reveals that supplementary psychotherapy can reduce depressive symptoms and increase remission rates in patients with TRD. Additionally, neurostimulation therapies - such as electroconvulsive therapy (ECT), transcranial magnetic stimulation (TMS), theta-burst stimulation, and magnetic seizure therapy - are used effectively in TRD treatment [5-9].

Managing TRD is complex and often frustrating for patients, who typically undergo multiple treatments without achieving remission. For example, ECT may require up to 12 sessions over four weeks [9]. While up to 70% of TRD patients are responsive to ECT, this leaves over a quarter of patients who do not respond [10]. What’s worse, an initial improvement after treatment does not guarantee full recovery, as many depressed patients relapse. Side effects - such as antipsychotic induced insomnia, antidepressant related weight gain, and lithium toxicity - frequently lead to nonadherence [11]. There is a need for new, effective, and tolerable therapies for TRD, with esketamine emerging as a promising candidate [12].

The U.S. Food and Drug Administration approved esketamine on March 5, 2019, for adults with TRD [12]. Early clinical evidence shows that this drug is effective as an adjunctive therapy for patients with TRD. The antidepressant effects of esketamine can be rapid in onset, and the responses may persist for months with proper medication administration. Indeed, esketamine treatment has repeatedly been associated with significant improvements in patient condition. This includes lowering patient Montgomery-Åsberg Depression Rating Scale scores [13, 14] and mitigation of suicidal ideation [15]. Long-term adjunctive esketamine therapy reduces rates of relapse compared to the use of first-line antidepressants alone [14].

Esketamine holds promise for addressing an unmet medical need with its novel mechanism of action and rapid onset of antidepressant effects. Unlike many other antidepressants that target the serotonergic or adrenergic systems, esketamine acts on N-methyl-D-aspartate (NMDA) receptors within the glutamatergic system [15]. Research also suggests that esketamine, uniquely, interacts with the opioid receptor system [15], which may contribute to its effectiveness in patients who are unresponsive to traditional medications. However, this mechanism also presents a risk of psychological dependence, especially given the similarity between esketamine and ketamine - a substance long associated with recreational drug use among young adults [16]. Although rates of ketamine abuse and addiction are relatively low compared to those of other recreational drugs [17], it is classified as a Schedule III substance by the Drug Enforcement Administration [18], indicating a “moderate to low potential for physical and psychological dependence.” Consequently, esketamine requires administration through a restricted distribution system under the Risk Evaluation and Mitigation Strategy [12].

The following case series aspires to give additional qualitative information on the effectiveness of esketamine by recounting the treatment course for a small number of TRD patients treated at a large suburban medical center in New Jersey (Jersey Shore University Medical Center, part of the Hackensack-Meridian Healthcare Network). These patients were trialed on esketamine, and their course was documented. Patients were selected due to previous failure of treatment via multiple pharmacological and procedural methods, including ECT. These are patients with TRD who were willing to be trialed on a novel medication and documented over time as a convenience sample for this study.

## Case presentation

Case 1

The first patient was a 52-year-old married Caucasian man with a 30-year history of generalized anxiety disorder, panic disorder, and depression. At his consultation for esketamine, he reported persistent fatigue, poor sleep, anhedonia, and a lack of interest in any activities, including household chores. Additionally, he experienced vivid nightmares related to his childhood emotional abuse. He also reported significant emotional distress stemming from constant worry about his life and future. He denied any suicidal ideation.

Before this consultation, his treatment regimen included vilazodone 40 mg, bupropion extended-release 300 mg, mirtazapine 15 mg, and L-methylfolate 15 mg, with no apparent symptomatic relief. He was also administered gabapentin 800 mg three times daily and lamotrigine 100 mg once daily; however, these were discontinued to facilitate ECT treatment. He underwent 10 ECT sessions over three weeks without improvement in depressive symptoms. He has also previously completed 56 sessions of repetitive TMS, which likewise produced no symptom improvement.

Previous pharmacological trials included sertraline 100 mg, fluoxetine 40 mg, paroxetine 40 mg, escitalopram 20 mg, extended-release venlafaxine 300 mg, duloxetine 60 mg, brexpiprazole 2 mg, and aripiprazole 10 mg; however, the patient exhibited no response to any of these psychotropic medications. He also underwent acupuncture and neuro-linguistic programming and reported a history of multiple inpatient psychiatric admissions.

The patient exhibited minimal improvement over the first three esketamine treatments but reported double vision and feeling "drunk" during the first 5-7 minutes of administration. His blood pressure also spiked, with systolic and diastolic pressures increasing by 20 mmHg and 10 mmHg, respectively. He experienced noticeable improvement in the first two to three days after each treatment, but this effect diminished significantly before the next weekly session. After completing 17 treatments, he decided to discontinue further treatment due to side effects and lack of sustained improvement.

Case 2

The second patient was a 25-year-old single Caucasian man with a history of anxiety and depression since early childhood. He presented with difficulty in falling and staying asleep, inability to enjoy life, poor concentration, low energy, hopelessness, and helplessness. His pharmacologic regimen at the time consisted of lithium 300 mg twice daily, trazodone 200 mg nightly, and risperidone 1 mg twice daily, with no symptom relief. He previously underwent 15 ECT sessions with no improvement.

The patient subsequently completed 12 esketamine treatments. He experienced dizziness and shakiness shortly after each treatment, although these side effects typically resolved within 30 minutes. During the course of treatment, he appeared brighter, more interactive, and participated more in household activities, leaving the house more frequently. His mother confirmed his improvement. However, the patient himself reported no subjective improvement and chose to discontinue treatment after 12 sessions.

Case 3

The third patient was a 73-year-old married Caucasian man with two adult children and a 20-year history of major depressive disorder. He was referred to the clinic by his primary psychiatrist for TRD. He reported poor sleep, low energy, impaired concentration, anhedonia, and severe anxiety, along with feelings of worthlessness, hopelessness, and a desire to give up. His current medications, fluvoxamine 200 mg daily and clonazepam 0.5 mg daily, provided no relief. The patient previously underwent 13 ECT sessions, which were also ineffective in alleviating his depression symptoms.

After the first three esketamine treatments, he felt less depressed, his anxiety was more manageable, and his anhedonia, his major concern at the presentation, was almost fully resolved. However, he experienced severe side effects during the following two treatments, including intense dizziness, unsteadiness, and a floating sensation that lasted up to four hours post-treatment. These side effects resulted in a relapse of his anxiety and depression symptoms, prompting him to discontinue the esketamine therapy.

Case 4

The fourth patient was a 37-year-old Caucasian woman with a history of depression, anxiety, mitochondrial myopathy, scoliosis, and back pain owing to herniated discs. She was referred by her psychiatrist for evaluation and treatment of her TRD. Over the past two weeks, she reported feeling hopeless and helpless, crying multiple times a day. Although she denied current suicidal ideation, she expressed that if it were not for her children, she would probably want to die. She stated, "I would stay in my house 24 hours a day if I could," and also endorsed confusion, increased word-finding difficulty, and decreased energy. Her symptoms had acutely worsened four years prior to presentation after the loss of her full-term baby, a trauma that caused significant guilt. At the time of presentation, she was administered extended-release bupropion 300 mg daily, vortioxetine 10 mg daily, fluoxetine 80 mg daily, clomipramine 75 mg daily, and clonazepam 2 mg twice daily, with no symptom relief. She also described feeling as though she were "walking through quicksand" due to her lack of energy and psychomotor retardation. To address this, she was prescribed mixed amphetamine salts 30 mg twice daily and an additional 30 mg of extended-release mixed amphetamine salts twice daily. Finally, she was administered zolpidem 10 mg nightly to assist with sleep, which she reported was effective. She also failed a full course of ECT.

The patient gradually responded to esketamine over the first eight treatments, reporting increased concentration and energy, and she was able to complete household chores. She became more involved in caring for her children and experienced improvement in her interpersonal relationships. By the completion of 12 treatments, she observed significant improvement overall with no major side effects reported. She opted to continue with maintenance esketamine treatment at a dose of 56 mg once weekly.

## Discussion

We selected the four cases described above from our TRD clinic. Patients were tried on esketamine therapy due to reporting a significant decrease in subjective quality of life due to depression that was refractory to two or more antidepressant medication regimens, as well as ECT. 

Although all four patients initially reported improvement with esketamine - with three patients reporting significant improvement - only one chose to continue with long-term treatment. The other three discontinued due to side effects, including double vision, dizziness, blood pressure spikes, and shakiness. While each patient experienced a unique side effect profile, Cases 1-3 all reported some form of significant dizziness. Despite mitigation of depression symptoms, the side effects were deemed by these patients as more disruptive than the TRD symptom relief was beneficial. Additionally, all three patients who discontinued treatment had longstanding depression diagnoses. The patients in Cases 1 and 2 had experienced depressive symptoms since childhood, while the patient in Case 3 had a 20-year history of depression. Given their extensive histories of failed treatments and the overall low recovery rates for TRD patients [10], a low success rate is not unexpected. This may also reflect the documented suspicion that there is a correlation between esketamine failure and previous ECT failure in patients with TRD [19]. An additional point of interest is that Case 3 reported improvement in anxiety symptoms despite the stimulant effects of esketamine (which are typically more pronounced at lower doses) [15].

The patient who chose to continue treatment (Case 4) should be further discussed. She also had previously failed multiple treatments, including several rounds of ECT. However, her symptoms had only worsened gradually over the past four years, and her psychiatric presentation was milder than that of the others. She was the only female patient and the only patient to exhibit symptoms of psychomotor retardation. Her social history was also unique, as her depression was compounded by her additional, extensive medical history and by grief following the loss of her full-term baby. These distinct features may have contributed to her positive response to esketamine, making them valuable topics for future empirical studies and clinical trials. However, it is also possible that the lack of reported physical side effects from esketamine was the primary contributor to long-term adherence.

While seeing only one out of four patients improve may be discouraging, this positive outcome is significant in the scope of TRD treatment. ECT is considered a more aggressive therapy for TRD, so any success in patients who have failed such treatment is an encouraging development [3, 5, 9].

Esketamine shows promise as a treatment option for TRD, including in patients who have not responded to ECT. However, patient selection should be cautious, with a thorough review of potential risks and side effects. This includes non-fatal effects such as dizziness, which can lower patient quality of life. This report documented two cases of patients discontinuing drug therapy due to such effects, despite reporting significant improvement in their depression. While adverse drug reactions should be handled with care and caution, it has been documented that a significant portion of patients experiencing side effects will see them mitigate with long-term use [20]. Therefore, patients should be thoroughly educated on common experiences of those taking esketamine and given coping strategies as an alternative to medication discontinuation, as appropriate. 

Results of formalized, large cohort drug trials have been mixed, with some showing high treatment efficacy and tolerability [21] while others warn of severe side effects, including increased suicidality [22]. However, those with TRD experience significant symptom burden, including suicidality, making this a tolerable-but-fearsome risk. Furthermore, side effects such as addiction and dissociation are widely reported but not experienced by a majority of patients [23]. Hopelessness is a significant component of depression, and it is often worsened for patients who experience a lack of improvement despite several rounds of pharmacological and procedural treatment [24]. Therefore, drugs like esketamine represent a lifeline for some patients who otherwise would have no means of effective symptom control.

Future studies should focus on establishing patient traits that act as predictors for esketamine success or failure. Notably, some debate currently exists around whether or not female patients respond better to esketamine [25]. This is a speculation that this case study reflects, as Case 4 (the only case resulting in sustained symptom improvement with long-term therapy tolerance) followed a female patient. Furthermore, this case series has made a novel observation in that Case 4's depression was the only one characterized by grief. Grief-related TRD having a higher response rate to esketamine is not an idea that has been tested in the literature, but it may have potential for future studies. 

Study limitations

As with all case studies, this report is limited by its small sample size and the differing circumstances of our four patients. It was also limited by low treatment adherence, which kept this study from following the cases over a longer period of time. 

## Conclusions

Esketamine is not a miracle drug and brings significant risks, including addiction, suicidality, and overdose. It has great potential but should be used cautiously in future trials, with mindfulness of its risks and uncomfortable side effects. It should be noted that some side effects of esketamine, especially addictive potential and dissociation, are widely reported but not experienced by a majority of patients. Understanding and treating TRD represents one of psychiatry's greatest current challenges. TRD is a condition with high clinical prevalence that comes with high levels of clinical burden, including longer hospital stays and more significant mortality risk. Perhaps most important is establishing unified diagnostic criteria and treatment guidelines for TRD, which is currently not a standalone condition in either the ICD-11 or DSM-5. More clear guidelines would help organize discussions in the literature as well as provide more clarity for providers during patient interactions. These guidelines would do well to include esketamine as a valid option for pharmacological treatment, with increasing nuance as more studies come out.
